# Multisector nutrition gains amidst evidence scarcity: scoping review of policies, data and interventions to reduce child stunting in Afghanistan

**DOI:** 10.1186/s12961-020-00569-x

**Published:** 2020-06-11

**Authors:** Christine Kim, Ghulam Farooq Mansoor, Pir Mohammad Paya, Mohammad Homayoun Ludin, Mohammad Javed Ahrar, Mohammad Omar Mashal, Catherine S. Todd

**Affiliations:** 1 Department of Health Policy and Management, Gillings School of Global Public Health, University of North Carolina at Chapel Hill, Chapel Hill, NC United States of America; 2 FHI 360/Integrated Hygiene, Sanitation, and Nutrition (IHSAN) project, Kabul, Afghanistan; 3 Public Nutrition Directorate, Ministry of Public Health, Islamic Republic of Afghanistan, Kabul, Afghanistan; 4 Rural Water Supply and Irrigation Programme (RuWATSIP) Department, Ministry of Rural Rehabilitation and Development (MRRD), Islamic Republic of Afghanistan, Kabul, Afghanistan; 5 Division of Reproductive, Maternal, Newborn, and Child Health, Global Health, Population and Nutrition Department, Durham, North, Carolina United States of America

**Keywords:** child nutrition, Afghanistan, water, sanitation and hygiene, nutrition policy, stunting, micronutrients, fragile and conflict-affected, multisectoral approach

## Abstract

**Background:**

Child health indicators have substantially improved across the last decade, yet Afghanistan has among the highest child stunting and malnutrition rates in Asia. Multisectoral approaches were recently introduced but evidence for this approach to improve support for and implementation of child nutrition programmes is limited compared to other countries.

**Methods:**

We reviewed policy and programme data to identify best practices and gaps surrounding child malnutrition in Afghanistan. We conducted a scoping review using broad search categories and approaches, including database and website searches, reference hand-searches, purposive policy and programme document request, and key informant interviews. Inclusion and exclusion criteria were developed iteratively, with abstracts and documents assessed against the final criteria. We abstracted documents systematically and summarised and synthesised content to generate the main findings.

**Results:**

We included 18 policies and strategies, 45 data sources and reports, and 20 intervention evaluations. Movement towards multisectoral efforts to address malnutrition at the policy level has started; however, integrated nutrition-specific and nutrition-sensitive interventions are not yet uniformly delivered at the community level. Many data sources capturing nutrition, food security and WASH (water, sanitation and hygiene) indicators are available but indicator definitions are not standardised and there are few longitudinal nutrition surveys. Political will to improve household nutrition status has shown increased government and donor investments in nutrition-sensitive and nutrition-specific programmes through combined small- and large-scale interventions between 2004 and 2013; however, evidence for interventions that effectively decrease stunting prevalence is limited.

**Conclusions:**

This review shows a breadth of nutrition programme, policy and data in Afghanistan. Multisector approaches faced challenges of reaching sufficient coverage as they often included a package of food security, livelihoods and health interventions but were each implemented independently. Further implementation evidence is needed to aid policy and programmes on effective integration of nutrition, food security and WASH in Afghanistan.

## Background

Globally, malnutrition affects one in three people and is a primary driver of the global burden of disease with high associated economic costs [1]. Africa and Asia lose 11% of their gross domestic product annually to malnutrition and fragile health systems are further strained by poor child health and development, micronutrient deficiencies, and nutrition-related infectious and non-communicable diseases [1, 2]. International partners and countries have increasingly recognised the global challenge of malnutrition and commitments to collaboratively end malnutrition in all forms have coalesced. In 2013, the *Lancet* published its second series on maternal and child malnutrition, highlighting the lack of high-quality evidence for and multisectoral inclusion of nutrition interventions as well as a lack of incorporation of nutrition-sensitive approaches such as water, sanitation and hygiene (WASH), agriculture, and food security into existing nutrition-specific programming [3]. The second Sustainable Development Goal (SDG) aims to “*end hunger, achieve food security and improved nutrition, and promote sustainable agriculture*” and 12 of 17 SDG indicators address some aspect of nutrition [4]. Thus, investments in nutrition are important to achieving the SDGs.

Malnutrition, specifically including fetal growth restriction, child stunting and wasting, and deficiencies of vitamin A and zinc, along with suboptimal breastfeeding, causes 3.1 million child deaths or almost 50% of all deaths under age 5 years annually [5]. The prevalence of malnutrition is significantly higher among fragile and conflict-affected settings [6]. Almost all countries ranked within the highest 10% of the Global Hunger Index [7] and with the highest stunting rates are classified as fragile or conflict affected [8]. Conflict and fragility affect nutritional status through different pathways and exacerbate poor health outcomes [9, 10]. Nearly 40% of the world’s stunted children live in South Asia [11]. Malnutrition is an indicator of social and political instability as it represents a multifaceted problem linked to poverty, food insecurity, and poor hygiene and health.

However, there is a growing evidence base of effective high impact interventions to reduce preventable malnutrition during the critical developmental window between pregnancy to 24 months of age [12]. These interventions include nutrition-sensitive components, such as WASH programmes, with better associated outcomes than nutrition-specific interventions. Poor sanitation, water quality and hygiene practices increase the risk for diarrheal disease and prevent adequate nutrient absorption in children. Diarrheal diseases are the second leading cause of death in children under 5 years of age, accounting for one in nine child deaths globally [13, 14]. Most (88%) diarrhoea-associated deaths are attributable to unsafe water, inadequate sanitation and insufficient hygiene [15]. Hand washing counselling can lead to a 30% reduction in diarrhoea [16]. Since 2014, the inter-relatedness of sanitation and nutrition has been elevated to a global policy priority. Existing evidence supports at least three direct pathways linking poor WASH access to a child’s nutritional status: diarrheal disease, intestinal parasite infections and environmental enteropathy [17]. Although the results of the Sanitation Hygiene Infant Nutrition Efficacy (SHINE) Trial and WASH Benefits Trial found that household-level WASH interventions were unlikely to reduce stunting or diarrhoea, they did not focus on community-wide coverage to achieve an open defecation-free status [18, 19]. Open defecation continues to be a challenge in Afghanistan and remains a community-level pathway to faecal contaminant exposure [20].

Afghanistan is the only country in the South Asia region in a conflict-affected and fragile situation [8, 21]. Afghanistan also ranks eighth on the Global Hunger Index, 14th for stunting rate and 42nd of 45 countries by the Hunger and Nutrition Commitment Index on country government’s political commitment to tackling hunger and malnutrition [1, 7, 22]. Globally, while investments have been made in implementing nutrition programmes and generating robust evidence, peer-reviewed data are limited and programme knowledge, often within the grey literature, is not widely shared or transferred. A 2018 series on nutrition in South Asia included two reviews, one on maternal nutrition interventions and another on optimal breastfeeding interventions. No studies from Afghanistan were eligible for inclusion based on the criteria for these reviews [23, 24]. However, there is a need to understand the available nutrition data from Afghanistan to improve knowledge sharing, evidence generation, policy development and programme implementation in the country and in other fragile settings facing similar challenges.

While other scoping reviews focus on evidence-based nutrition interventions, nutrition-related data sources and policies contextualise evidence generation and prioritisation. This scoping review therefore maps nutrition-related policies, data and interventions aiming to reduce child stunting in Afghanistan and the extent to which multisectoral involvement contributed to their success. The objectives were to (1) delineate nutrition policy and programmes and the degree of multisectoral inclusion; (2) describe the scope of available published and grey literature; and (3) identify programme and knowledge gaps. We explore nutrition-related policy, data and evidence sources to make summary recommendations to inform multisectoral efforts to reduce child malnutrition in Afghanistan.

Box 1 Inclusion and exclusion criteria Inclusion criteria

Peer-reviewed article

- Peer-reviewed research on nutrition, food security and/or WASH interventions
- Includes a measure of at least one child nutrition outcome or nutrition-related knowledge or behaviour (includes hygiene)
- Specific to Afghanistan

Grey literature

- Programmatic research or evaluation done by third party
- Explicit interventions/programmes on nutrition, food, security and/or WASH with expected changes in nutritional status
- Implemented in Afghanistan

Data source

- Measurement of nutrition-related knowledge, behaviours or outcomes
- Data collected from Afghanistan

Policy document

- All relevant government policies included

Exclusion criteria

- Not publicly disseminated or unavailable to public upon request from source
- Not specific to Afghanistan
- Target population is Afghan refugees no longer in the country or children older than 5 years
- Not written in English
- Global or multi-country study without specific data from Afghanistan, rather only aggregated global or regional estimates
- Agriculture, food security, economic development, or water and sanitation-related programming and/or research without explicit child nutrition component or measures

## Methods

### Study design

We conducted a scoping review using the six-stage methodological framework proposed by Arksey and O’Malley [25] and enhanced by Levac et al. [26]. The stages include (1) identifying the research question, (2) searching for relevant sources, (3) selecting sources, (4) extracting data, (5) collating, summarising and reporting, and (6) consulting stakeholders [25, 27]. We selected a scoping exercise rather than a systematic review to map the wide range of published and grey literature, policy and data sources on child stunting in Afghanistan and to better define the gaps within existing practice and research. Additionally, we applied a two-step process to iteratively identify and select sources as well as to engage stakeholders [26]. The first step comprised a rapid desk review with a broad literature review and stakeholder interviews conducted in August 2017 to understand available sources of and gaps in information. A second systematic literature search and expanded hand-search of relevant content was conducted between September 2018 and February 2019. We followed the PRISMA checklist for scoping reviews. No scoping review protocol was registered publicly for this study.

### Search strategy

The initial literature search was conducted through MEDLINE/PubMed and Google Scholar, with the following search terms: ((“nutrition sensitive” OR “nutrition specific” OR “WASH” OR “water sanitation hygiene” OR “food security” OR “nutrition” OR “infant and young child feeding” OR “IYCF” OR “maternal nutrition”) AND “Afghanistan”). Websites for Afghan government ministries, non-government organisation (NGO) implementing partners in the health sector and donors were purposively hand-searched for relevant reports. Key informants in the government (Ministry of Public Health (MoPH), Ministry of Rural Rehabilitation and Development, and the Ministry of Agriculture, Irrigation and Livestock (MAIL)), NGOs, members of nutrition-related technical working groups, and provincial health officials were interviewed in person or contacted with an email-based electronic survey. Stakeholders were engaged initially to understand their past and current nutrition, food security and/or WASH programming as well as the data sources and evidence used to inform their policies and programmes.

A second literature search was conducted across 13 databases, namely MEDLINE/PubMed, Popline, Embase, Global Health, Academic Search Premier, CINAHL Plus with Full Text, EconLit, Education Full Text (H.W. Wilson), Environment Complete, ERIC, GreenFILE, Middle Eastern and Central Asian Studies, and Web of Science. Example search terms included (from PubMed): (“child nutrition” OR “infant and young child feeding” OR child nutrition sciences [Mesh] OR “infant and young child feeding” OR Prenatal Nutritional Physiological Phenomena [Mesh] OR child nutritional physiological phenomena [Mesh] OR ((nutritional status [Mesh] OR “nutrition sensitive” OR “nutrition” OR nutritional requirements [Mesh] OR “nutrition specific” OR “undernutrition” OR “malnutrition” OR hunger OR nutrition assessment [Mesh] OR nutrition surveys [Mesh] OR nutrition policy [Mesh] OR “nutrition during pregnancy” OR “maternal nutrition” OR “maternal undernutrition” OR “maternal malnutrition” OR “Maternal Nutritional Physiological Phenomena” [Mesh] OR “maternal iron supplementation” OR “dietary supplements during pregnancy” OR Food Labeling [Mesh] OR “exclusive breastfeeding” OR “feeding practice” OR dietary diversity OR MUAC OR “mid-upper arm circumference” OR stunting OR wasting) AND (child OR children OR infant)) AND Afghanistan [Mesh]). Table S1 contains the complete search strategy for each database. Reference lists were hand-searched for relevant sources from 14 review papers identified through database searches. A purposive hand-search was then conducted for grey sources from websites of government ministries, NGO implementing partners in the health sector and donors.

### Source selection

Prior to selecting sources, we agreed on broad inclusion and exclusion criteria and used these at the first search stage. We then refined these criteria in an iterative process during the first phase of abstract review, which delineated relevant content and sources [26]. This approach helped alleviate uncertainties surrounding source selection, given the broad research question and goal of including grey literature sources.

To map the current child nutrition situation in Afghanistan, we categorised our sources into published articles, grey literature reports, data sources and policy documents. Final inclusion and exclusion criteria are shown in Box 1.

All relevant data sources and policy documents were included for analysis. Only published articles and grey literature underwent full text screening. Two independent reviewers screened all abstracts/titles (CK and CT) and selected full texts (GFM and MOM). Any disagreements on source inclusion were resolved by the two reviewers and, when necessary, by a third reviewer. We did not appraise source quality as we aimed to include all relevant sources to provide a full landscape of national nutrition programming.

### Citation management and data extraction

All citations were imported into Endnote X7 (Clarivate Analytics, PA, United States of America). Duplicate citations were removed manually during screening. Data were extracted into a Microsoft Excel spreadsheet. Descriptive information from each source was extracted by specified categories. Table 1 lists the data elements extracted for each type of source.

**Table 1:** Data extraction elements from sources

Types of source	Extraction categories
Published peer-reviewed studies and grey literature reports	Author(s), publication year, title, source type (published or grey), product of database search or hand search, objectives, study design (research, evaluation, case study), location of study (national, sub-national), population (households, children <5 years, children 0–24 months), sample size if applicable, nutrition-related outcomes measured, multi-sectoral approach if applicable, enabling environment, and lessons learned
Data sources	Citation, description, year of publication, primary or secondary analysis, sample size, geographic coverage, child nutrition-related measures, level of summary of evidence for decision-making and biases in sampling/results interpretation
Policy documents	Citation, year, government agency, department, multisectoral approaches, child nutrition-related objectives, main child nutrition-related strategies, changes from last iteration and programmatic responses

### Analysis

We first collated and summarised the scope of available sources and then described the nutrition programmes that had been evaluated and the extent to which they contained multisectoral approaches. We synthesised information identifying changes to priority areas and outcomes over time in data sources and policy documents, respectively, with iterative identification of programme and knowledge gaps. Only the key findings are presented in this paper.

## Results

### Scope of sources

**Fig. 1:**
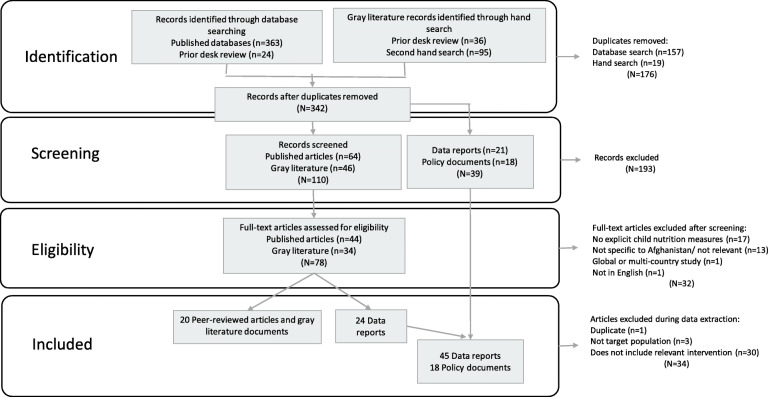
Flow diagram of source selection

After removing duplicates from a combination of database, prior desk review and a second hand-search results, 342 source documents were screened by abstract and title. All relevant data reports and policy documents were included, while full-text screening was conducted for 64 peer-reviewed articles and 46 grey literature documents. We classified 25 studies as data reports because they included secondary data analyses of nutrition outcomes and formative research or provided other relevant nutrition-related evidence but did not include an intervention. Overall, we found 46 data reports, 18 policy documents and 20 full text studies (peer-reviewed articles and grey literature programme reports) that met the inclusion criteria for this scoping review (Fig. 1).

We first provide an overview of government policy responses to child malnutrition. Next, we present the ways in which nutrition status and nutrition-related indicators have been measured and studied in Afghanistan. Finally, we present nutrition-specific and nutrition-sensitive interventions that aimed to improve the nutrition status of children.

### Policy responses to child malnutrition

**Fig. 2:**
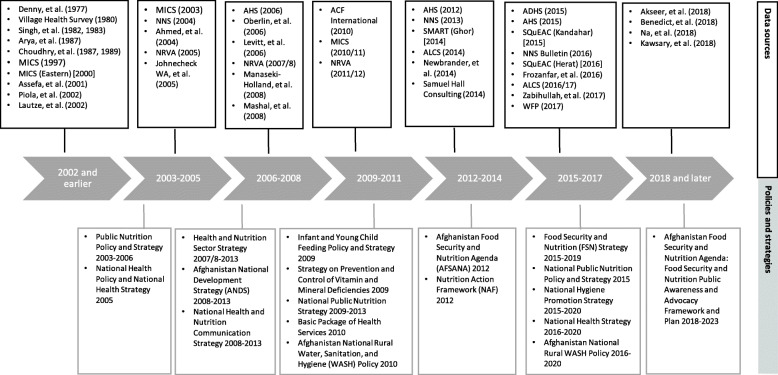
Timeline of data sources and policies

Multiple policies and strategies have been developed over the past 15 years at the level of government ministries to address nutrition, WASH and food security. More recently, government-wide, multisectoral policies have been introduced to coordinate and empower ministries to collaboratively work towards national nutrition and food security goals. Figure 2 illustrates the timeline for policies and data/evidence published around nutrition, food security and WASH included in this review. We identified 18 relevant policies and strategies that incorporated the nutrition objectives between 2003 and 2018. The first Public Nutrition Policy and Strategy was developed in 2003, with follow-up documents in 2009 and 2015. These policy and strategy documents largely continue the same objectives, with more specific strategies described in the latter two periods such as targeting specific vulnerable populations like pregnant women, adolescent girls and children under 5 years of age. Addressing malnutrition has consistently been a key objective of the health sector, present in both the National Health Policy and National Health Strategy since 2005. In 2009, two specific strategies were developed to address poor infant and young child feeding (IYCF) practices and prevent and reduce micronutrient deficiencies. While these health sector policies and strategies acknowledged multisectoral pathways of malnutrition and the need for coordination between line ministries for effective nutrition-sensitive intervention programming, engagement of non-health sector ministries in nutrition-related actions did not occur until after 2010. This is possibly due to the influence of the Afghanistan National Development Strategy (ANDS) in 2008, which was the country’s overall strategy for security, governance, economic growth, social development and poverty reduction to reach the Millennium Development Goals. The ANDS outlined the country’s development policies and was the first extensive multisector strategy endorsed by the government and international partners. While the ANDS provided an overall country strategy towards development, including nutrition, food security and WASH actions, government-wide efforts to recognise nutrition as foundational to development, establish nutrition targets for which all related sectors are responsible, and identify feasible actions to achieve nutrition targets were only initiated in 2012.

While nutrition requires multisectoral actions, in the Afghanistan context, it is more realistic for institutions to initially build capacity by sector because integrated or multisectoral service delivery requires stronger institutional capacity [2]. Institutional capacities required to effectively mount a multisectoral response to malnutrition include sufficient capacity to undertake policy-making, programme design, implementation, training, monitoring, and changing perceptions and understanding malnutrition [28]. Two key documents developed in 2012 established nutrition as a government priority, the Nutrition Action Framework and the Afghanistan Food Security and Nutrition Agenda (AFSANA, changed to AFSeN in April 2017). AFSeN provides the government’s policy statement affirming its commitment and determination to have a coordinated response to the underlying determinants of hunger and malnutrition. While it is a comprehensive policy document, there has been relatively low operationalisation to date by line ministries. By 2015, three of the five key nutrition, food security, and WASH-related policy and strategy documents were developed with government-wide support between the MoPH, Ministry of Rural Rehabilitation and Development, MAIL, and the Ministry of Education [29–31]. In 2018, an advocacy and information strategy and an implementation plan were developed as a key step toward AFSeN strategy functionality [32]. The plan was developed by a multi-agency working group comprising representatives from the MoPH, MAIL, Ministry of Education, Central Statistics Office, Ministry of Information and Cultural Affairs, Ministry of Women’s Affairs, Ministry of Energy and Water, Afghan National Standards Authority, Afghanistan Human Rights Organization and international partners.

All policy and strategy documents referenced the most recent descriptive statistics, relying heavily on the 2013 National Nutrition Survey (NNS) and the 2017 National Risk and Vulnerability Assessment (NRVA)/Afghanistan Living Conditions Survey (ALCS), but less commonly referenced peer-reviewed statistical analyses of predictors of nutrition outcomes. There was a focus on using national level statistics, even when disaggregated provincial or regional statistics were available. While variation in nutrition status across provinces was recognised and the need for context-specific strategies was often acknowledged, provincial or regional estimates were not cited or used for strategy development or for evaluating changes in nutrition status over time. Recent policies and strategies emphasised and defined multisectoral inclusion by the line ministry, especially when discussing intervention strategies across sectors and coordinating among ministries and implementing partners. However, the integration of intervention strategies was less detailed in these documents. Programme strategy responses between 2004 and 2009 focused on the treatment and prevention of malnutrition through health service delivery, control of micronutrient deficiencies through supplementation, and IYCF practices. While social and behaviour change programming, treatment guidelines and community-based nutrition services (e.g. management of moderate acute malnutrition (MAM), community-based management of acute malnutrition (CMAM) pilots and integrated community case management) were introduced prior to 2013, the more recent strategies focused on continued quality improvement, integrated strategies (integrated management of acute malnutrition (IMAM) in 2014), and taking these services to greater scale [33]. Additionally, food quality, safety and standards, and expanding nutrition-sensitive food products in agriculture programmes were frequently mentioned, though linkages to health programmes were limited. Further, it was unclear whether integrating nutrition with other health programmes occurred at the implementation level because, while strategies highlighted multipronged or multisectoral approaches, they were not explicit regarding the level of integration. Table 2 provides a summary of all relevant policies and strategies.

**Table 2:** Summary of policy documents

Year	2004–2005	2006–2007	2008–2009	2010–2013	2014–2015	2016–2018
Government agency (number of policies)	MoPH (2)	MoPH (1)	MoPH (4) Government (1)	MoPH (1) MRRD (1) Government (2)	MAIL (1) MoPH (2)	MoPH (1) MRRD (1) Government (1)
Multisectoral considerations	Both recognise multi-causal nature of malnutrition and need for MoPH collaboration with other ministries	MoPH policy acknowledges need for broad-based interventions to tackle malnutrition, specifically for nutritious foods and education/awareness but largely remains health sector focused	MoPH policies similar to previous years; ANDS is multisector by design and guides overall development strategy	MoPH BPHS guidelines are health service delivery specific; WASH Policy, AFSANA and NAF address multisectoral approaches for improving nutrition outcomes with WASH and food security	Public Nutrition Policy and FSN have extensive linkages to food security and safety strategies and their effect on nutrition; Hygiene Promotion Strategy covers sanitation, personal hygiene and food hygiene messages	MoPH National Health Strategy has a strong health sector focus; AFSeN was developed across multiple line ministries, including nutrition and food security
Main nutrition objectives	Ensure prevalence of acute malnutrition or wasting remains <5% for children under 5 years old; access to iodised salt; control micronutrient deficiency disease outbreaks; increase EBF; increase public nutrition capacity and skills	Increase service coverage and quality to prevent and treat communicable diseases and malnutrition among children and adults	New content includes increasing appropriate IYCF practices; reduce major micronutrient deficiency disorder prevalence; ANDS outlines government-wide efforts to recognise nutrition as development foundation, establish nutrition target responsibility across sectors and identify feasible actions to achieve targets	Deliver BPHS/EPHS nutrition component; improve access to safe drinking water, make communities ODF, increase hygiene awareness and practices; improve availability, access to and use of healthy foods	Largely a continuation of earlier objectives	Greater political and social commitment to improve food security and nutrition through increased financial resources; advocate for involvement of private and public sectors and communities in food security and nutrition activities
Key nutrition strategies	National food security and nutrition surveillance system; nutrition surveys with standardised indicators; household food security interventions; adequate nutritious food aid; emergency SFPs; universal salt iodisation; integrated micronutrient education, treatment, supplementation and food fortification; appropriate IYCF support and promotion; establish appropriate services for SAM diagnosis and treatment; nutrition education, communication and advocacy; integrated IMCI	In addition to previous strategies, collaborate with other line ministries to address environmental health consequences of poor water supplies and lack of adequate sanitation facilities	In addition to previous strategies, adopt public nutrition approach involving multi-sectoral interventions (food insecurity, poor social environment and inadequate health service access); focus on quality salt iodisation, flour fortification, diarrhoeal interventions and therapeutic feeding of hospitalised malnourished children; application of IYCF policy and strategy supported by advocacy, technical guidance and law enforcement; IYCF promotion and counselling implemented within BPHS and EPHS in all health facilities; public–private partnerships with food industry and local markets	In addition to previous strategies, hygiene education in schools, community groups and women’s groups; establish and maintain community water systems, community-led household latrine promotion and construction; increase food availability through production and dietary diversification, food storage and preservation, market availability; improve food access through food transfers, food for work or assets, poverty alleviation programmes and community-based income generation; National Solidarity Program/Citizen's Charter Program	In addition to previous strategies, multipronged approach to address micronutrient deficiency problems, with special focus on anaemia and iron deficiency among women of reproductive age and children 6–59 months of age	Advocacy to prioritise audiences through meetings, workshops and seminars, with nutrition advocacy materials package for each target audience to build a critical mass of food security and nutrition advocates and promote a national coordinated effort to improve food security and nutrition; CLTS
Changes over time	Baseline nutrition policy and strategy	Language more specific to environmental factors and linkages beyond health sector, nutrition IEC, service provision and training; no nutrition indicators included in M&E plan for national policy/strategy	Identified target groups: women, adolescent girls and children; expanded target micronutrient deficiencies; mapping nutrition indicators by source; more comprehensive list of strategies linked to each objective	Emphasis on nutritious food programming and community-led WASH (with separate implementation from nutrition programmes)	No significant changes from previous years, rather continuation of identified key strategies	Prioritise advocacy audiences of multisectoral government ministries and authorities, private sector (e.g. food producers, importers and retailers), religious leaders, development partners, donors, civil society organisations, and media
Programmatic responses	Established Consultative Group for Health and Nutrition to coordinate work across ministries and donors; implemented public nutrition component within BPHS, including micronutrient supplementation, clinical malnutrition treatment, measles and vitamin A campaigns (coverage targets >95% and >85%, respectively); 25 iodised salt factories established through partnerships with private sector and MoM; small-scale flour fortification	Implemented nutrition services within EPHS with BPHS; 47 TFUs established within provincial hospitals; Piloted CMAM; SFP added in 2009 to CMAM pilot; Food and Drug Quality Control Department established in MoPH; Quality Control department in MAIL developed legislation, regulatory frameworks, standards, etc. on certification systems and laboratory testing for food quality and safety; Nutrition Cluster activated	IYCF public awareness campaigns; Baby Friendly Hospital Initiative, complementary feeding (recipes and participatory cooking sessions); passage of Maternity Protection Act; Code of Marketing of Breast Milk Substitutes adopted by government; application of the Positive Deviance-Hearth model; and piloting C-GMP; formative research on infant and young child feeding practices, including TIPS and recipes; developed breastfeeding counselling tools and trained 80 breastfeeding master trainers and 3000 counsellors in health facilities; Celebration of World Breastfeeding Week annually, launched National Breastfeeding Communication Campaign in 2009; introduction of re-lactation support as part of TFUs; refurbished MoPH equipment and labs; Afghan National Standards Authority established	Established Food and Nutrition Secretariat and high-level steering committee; efforts to implement nutrition-sensitive programmes increasing with improved HED capacity in MAIL and agriculture projects are designed to be more nutrition sensitive	Promotion of home-based food processing, storage and conservation, particularly for women; IEC on food and nutrition issues; food safety standards and control; expansion of nutrition sensitive products (vegetables, fruits) in home gardens and on agricultural land	CLTS aimed at supporting communities to be open defecation-free through hygiene education, community mobilisation and behaviour change

*AFSANA* Afghanistan Food Security and Nutrition Agenda, *AFSeN* Afghanistan Food Security and Nutrition, *ANDS* Afghanistan National Development Strategy, *BPHS* basic package of health services, *C-GMP* Community Growth Monitoring and Promotion, *CLTS* community-led total sanitation, *CMAM* community-based management of acute malnutrition, *EBF* exclusive breastfeeding, *EPHS* essential package of hospital services, *FSN* food security and nutrition, *HED* Home Economic Department, *IEC* information, education, communication, *IMCI* integrated management of childhood illnesses, *IYCF* infant and young child feeding, *MAIL* Ministry of Agriculture, Irrigation, and Livestock, *MoM* Ministry of Mines, *MoPH* Ministry of Public Health, *MRRD* Ministry of Rural Rehabilitation and Development, *NAF* Nutrition Action Framework, *ODF* Open defecation free, *SAM* severe acute malnutrition, *SFP* supplementary feeding programme, *TFU* therapeutic feeding units, *TIPS* Trial of Improved Practices, *WASH* water, sanitation, and hygiene

### Assessing the nutrition situation in children: data sources

#### Overall nutrition status

Stunting of children 0–59 months was 40.9% in 2013, with severe stunting at 20.9% and moderate stunting at 20.0% [34]. Stunting prevalence varied from 24.3% to 70.8% by province. While still high, these numbers represent a decrease in national stunting rates from 60.5% in 2004 [35]. Micronutrient deficiencies were also pervasive among women of reproductive age and children 6–59 months of age. There is evidence of low dietary diversity among all Afghans, particularly among children under 2 years of age. In 2015, just under a quarter of children under 2 years of age had been given foods from the minimum number of food groups and half were fed at least the minimum number of times appropriate for their age [36]. Overall, correct IYCF practices, particularly exclusive breastfeeding to 6 months and frequency of complementary feeds, remained low and there was a disparity between rural and urban areas in the percentage of children aged 6–23 months fed according to IYCF guidelines (13% vs. 22%, respectively) [36]. Only 58.9% of children under 5 years lived in households using iodised salt, which is far below the 90% recommendation for household coverage to eliminate iodine deficiency [36, 37].

Expansion of access to improved drinking water and sanitation facilities has been slow. In 2015, over one-third of the population did not have access to an improved source of drinking water and three-quarters did not have access to an improved sanitation facility [34, 36, 38]. In 2013, the percentage of women who reported handwashing (89.7%) exceeded the observed available handwashing places in the home (45.1%) [34].

#### Overview of key national data sources

Since 2002, nutrition data within Afghanistan has increased (Table 3). Overall, while more surveys have collected nutrition-related information from households since 2012, there has also been a significant increase in the analysis of population-based survey data to better understand the factors associated with poor nutrition practices and outcomes. Prior to 2004, evidence on child nutrition status was confined to small area surveys specific to informing small-scale health and nutrition programmes or hospital surveys based on disease-specific cases with malnutrition as a secondary outcome or additional risk factor for mortality. The first NNS [39] was conducted in 2004 and provided the only comprehensive national nutrition assessment; however, provincial level estimates were not available due to the sampling methodology. In 2013, another NNS was conducted to provide both national and provincial level representative estimates for key nutrition indicators, except for micronutrient-related deficiencies due to the limited sample collection. The 2013 NNS [34] also included a qualitative knowledge, attitudes and practices component on IYCF and on prevention of micronutrient deficiencies. We use the two NNS as time bounds to understand the generation of nutrition-related data before, during and after they were conducted, given the impact of these data on nutrition policy and programming (Table 3).

**Table 3:** Summary of data sources with nutrition-related measures

		2004 NNS and earlier	2005–2012	2013 NNS and later
**Number of data sources**		15	12	18
**Type of data collection**	Primary data collection reports	15	10	13
	Secondary data analysis	0	1	4
	Use of both primary and secondary data	0	1	1
**Geographic representation**	National	1	2	4
	National and/or Regional	2 (2 regional only)	1	1
	National and/or Provincial	0	3	5 (4 single-province/district/urban areas only)
	Specific area to programme/facility and/or not representative of any region	10	6	4
**Implementers**	NGO	11	3	6
	Government/CSO	1	4	4
	Academic institution/Research organisation (National or Int'l)	3	5	8
**Child nutrition-related measures**	**IYCF**			
	Ever breastfed	3	1	2
	Early initiation of breastfeeding	3	2	5
	Use of pre-lacteal feed	1	0	2
	Discarded colostrum	2	0	1
	Exclusive breastfeeding	3 m (1); 4 m (2); 6 m (1)	5 m (2); 6 m (2)	5 m (2); 6 m (4)
	Complementary foods	6–9 m (4)	6–9 m (4)	6–8 m (4); 6–9 m (1)
	Minimum acceptable diet (4+ groups)	0	0	2
	Minimum meal frequency	0	1	2
	Continued breastfeeding at 1 year	3	1	3
	**Micronutrients**			
	Vitamin A supplementation/deficiency/night blindness	4	6	4
	Iodised salts/visible goitre/iodine deficiency disorders	4	3	2
	Anaemia/iron deficiency	1	2	1
	Zinc deficiency	0	0	1
	Vitamin D deficiency	0	1	1
	Vitamin C deficiency	1	0	0
	**Immunisations**			
	Measles	4	5	3
	Fully immunized	2	2	2
	PENTA 3	0	1	1
	**WASH**			
	Safe drinking water	4	4	6
	Household water insecurity	1	0	0
	Improved sanitation	5	4	7
	Handwashing with soap/ash, at key times	1	1	5
	**Food security**			
	Sufficient food last week	1	0	0
	Household perception of food security	0	2	0
	Dietary diversity	0	1	3
	Calorie deficiency	0	1	1
	Protein deficiency	0	1	1
	Hunger scale	0	0	1
	Food insecure population	0	0	2
	Households receiving food aid	1	0	0
	Households owning garden plot	0	0	3
	Acceptable food consumption/diet, coping mechanisms	1	0	2
	**Childhood illness**			
	Diarrhoea in 2 weeks preceding survey	5	4	3
	ARI in 2 weeks preceding survey	4	3	2
	Other illnesses related to malnutrition or outcomes affected by malnutrition	Pyogenic meningitis (1) Measles (3)	0	0
	**Nutrition outcomes**			
	MUAC GAM/MAM/SAM	4	1	5
	Wasting/MAM/SAM (weight for height <-2 SD)	4	3	3
	Underweight (weight for age <-2 SD)	3	2	3
	Stunting (height for age <-2 SD)	4	3	3
	Overweight (weight for height >+2 SD)	0	0	1
	Low birth weight	0	0	1
**Sources without nutrition indicators**	Qualitative study	0	0	2
	Statistical analyses on variables associated with nutrition outcomes	0	0	4

*ARI* acute respiratory infection, *CSO* Central Statistics Organization, *GAM* global acute malnutrition, *IYCF* infant and young child feeding, *m* months, *MAM* moderate acute malnutrition, *MUAC* mid-upper arm circumference, *NGO* non-governmental organisation, *NNS* National Nutrition Survey, *PENTA* pentavalent vaccine (diphtheria, pertussis, tetanus, hepatitis B, *Haemophilus influenza*), *SAM* severe acute malnutrition, *SD* standard deviation, *WASH* water, sanitation, and hygiene

The Afghanistan Living Conditions Survey [40] (ALCS, previously known as the NRVA) was most recently conducted in 2016–2017 and provides multisectoral estimates on poverty, food security, education, health, labour market, agriculture and other critical indicators. The NRVA/ALCS has been implemented routinely, generally at 2–3-year intervals, since 2003. The Multiple Indicator Cluster Survey (MICS) was conducted four times, three of which were before 2004. The MICS is supported by UNICEF and focuses on child rights and development indicators. Two health sector-specific surveys are the Afghanistan Health Survey (AHS) [41] and the Afghanistan Demographic and Health Survey (ADHS) [42]. The AHS, implemented since 2006, is an annual survey that provides national and provincial level estimates on priority health sector indicators and is used to evaluate the health service delivery projects, most recently the System Enhancement for Health Action in Transition. The ADHS was first implemented in 2015 to provide national and provincial level estimates on a range of demographic and health indicators collected in other low- and middle-income countries. Programme-related rapid nutrition assessments have been conducted more recently (Standardized Monitoring and Assessment of Relief and Transitions (SMART) surveys [43–45] and Semi-Quantitative Evaluation of Access and Coverage (SQUEAC) [46, 47]). These assessments aim to provide rapid coverage estimates and programme area information on population nutrition status at the subnational level. All aforementioned surveys are cross-sectional in design. Finally, the national nutrition surveillance bulletin provides quarterly surveillance information across 175 facility and 953 community sentinel sites [48].

Survey implementation varied between government agencies (mainly the Central Statistics Office), academic or research institutions, and NGOs. Child nutrition-related measures are organised into eight categories, namely IYCF, micronutrients, immunisations, mid-upper arm circumference (MUAC), WASH, food security, childhood illness and nutrition outcomes. Duplication of data collection was minimal during each time period as no data sources collected identical nutrition indicators. We incorporated commonly reported nutrition-related indicators into the IYCF practices, immunisation and WASH categories.

#### Nutrition outcomes

Anthropometric measures have been used to estimate the prevalence of malnutrition from before 2002. These measures were obtained during household surveys and nutrition status was assessed using a MUAC as well as weight-for-height (wasting), weight-for-age (underweight) and height-for-age (stunting), though not all indicators have been used consistently across studies. The prevalence of the overweight population was not assessed until the 2013 NNS. MUAC was more widely used across national-level household surveys and among programme area assessments of nutrition status. Most data sources did not measure nutrition outcomes; only the MICS and NNS reported on wasting, stunting and underweight. Small area surveys reported global acute malnutrition and severe acute malnutrition estimates. Methodological difficulties capturing anthropometric data in this context compromised obtaining children’s precise age and height, including a representative sample of vulnerable populations, and limited reference data for infants less than 6 months old [49].

#### Micronutrient deficiencies

Vitamin A supplementation and presence of household iodised salt have been consistently monitored over time. Reported micronutrient deficiency data sources prior to 2004 were based on small-scale surveys and assessed clinical signs of diseases such as scurvy [50]. In the 2004 NNS, 81% of children aged 6–59 months received vitamin A supplementation and 28.3% of households were found to have any iodine in their salt [39]. Anaemia and iron deficiency anaemia were also measured; 37.9% of children aged 6–59 months were anaemic and 33.4% had iron deficiency. In the 2013 NNS, 44.6% of children under 5 years received vitamin A supplementation, 44.9% were anaemic and 13.7% had iron deficiency. The 2013 NNS also collected data for vitamin D (81%) and zinc deficiency (15.1%) in the same age group.

#### Infant and young child feeding practices

Evidence on optimal breastfeeding practices in Afghanistan shows decreasing prevalence of key IYCF indicators, including early initiation of breastfeeding within 1 hour of birth, exclusive breastfeeding (EBF) for the first 6 months, and continued breastfeeding up to 2 years of age with appropriate complementary foods [23]. These practices may have always been poor as documented by a 1983 study of infant feeding practices in Kabul [51]. This study captured the traditional practice of pre-lacteal feeds, such as butter or tea, early breastfeeding cessation due to perceived insufficient milk supply, and poor introduction of complementary foods [51]. Most data sources have not collected all IYCF indicators; rather, only one or two breastfeeding-related indicators are reported. Further, the age ranges used for EBF and continued breastfeeding measures have been inconsistent across sources [34, 36, 41, 52].

The transition from EBF to solid foods is a critical period for children as they are most vulnerable to becoming undernourished during this time. Minimum acceptable diet and minimum meal frequency are relatively new indicators that serve as proxies for adequate micronutrient density of foods and energy requirements, respectively. These have only been collected by recent large national health surveys. The 2015 ADHS found that only 16% of children aged 6–23 months received the minimum acceptable diet [36]. Qualitative studies describing IYCF practices and beliefs around infant feeding have only been conducted since the 2013 NNS. These studies highlighted traditional practices and regional preferences for pre-lacteal feeds and the rejection of colostrum often instructed by mothers-in-law [34], the perceived need to bathe an infant before initiating breastfeeding [53] and perceptions around low milk supply [34, 54].

#### Healthcare and environment

Indicators for immunisation (immunised for measles or fully immunised by 24 months), WASH (safe drinking water and improved sanitation) and childhood illnesses (diarrhoea and acute respiratory infection) were more widely collected across health and non-health data sources. The ADHS found that 46% of children were fully vaccinated, 65% of households had access to an improved drinking water source, 25% of households had access to improved sanitation facilities, and 29% of children had diarrhoea 2 weeks before the survey [36]. Since 2013, more data have been collected on caregiver (generally mothers) handwashing practices at critical times (47.5% of mothers) such as before feeding a child and after disposing of a child’s faeces [55]. One study found that, in urban areas, only 33% of respondents reported handwashing before eating and only 21% of female respondents reported handwashing before food preparation [56].

#### Food security and dietary diversity

Before 2013, there was less information available regarding underlying causes of malnutrition related to food security, livelihoods and dietary diversity. While efforts were made to integrate nutrition information into wider vulnerability assessments, such as the NRVA, food security indicators have not been consistently defined or collected. Food security and dietary diversity measures ranged across different sectors, with the food security situation predominantly described qualitatively. Overall poor access to diverse foods and low dietary diversity has been documented since before 2004; vulnerability to food insecurity was documented as a mixture of households’ ability to cope with economic risks, socio-political and geographic risks, natural and manufactured hazards, and risks due to food aid delivery [57]. The 2005 NRVA estimated that 24% of households had low dietary diversity and 44% perceived themselves to be food insecure. The 2014 ALCS estimated that 33% of the population was food insecure. These values are not comparable due to differences in indicator definitions. Levitt et al. [58] found that food availability is generally not the main concern in Afghanistan; rather, dietary diversity is limited due to poor economic ability to access a variety of available foods.

#### Secondary data analyses

Secondary data analyses have increased since the 2013 NNS as more national and provincial level representative sample surveys were conducted. These studies have assessed trends in IYCF indicators and associated factors [23], geographic disparities in nutrition status and factors associated with stunting and underweight [59], factors associated with poor complementary feeding practices [60], and the relationship between irrigation and dietary diversity [61]. Benedict et al. [23] found that all positive IYCF indicators have decreased significantly; for example, EBF significantly decreased by 10 percentage points from 2010 to 2016. Large district-level disparities exist in child nutrition outcomes such as stunting prevalence ranging from 4% to 84% [59]. A greater number of antenatal care visits and increasing child age were positively associated with meeting all complementary feeding practices [60]. Irrigation facilities were found to be important and positively correlated with diversity of food intake from households’ own production and garden plots were positively associated with greater diversity of food purchased at markets [61].

#### Presenting summary evidence for decision-making

The presentation of results and recommendations based on the generated data are important to guide policy-makers and other stakeholders to make evidence-based decisions. Survey reports generally included an executive summary with a key indicators table. Policy recommendations based on results differed among reports and most reports did not include their key recommendations in the executive summary. More recent studies have included trends in key indicators using several data points such as the AHS 2015 and the ALCS 2016–2017. However, most studies cautioned against comparisons with previous estimates due to differences in methodology and indicator definitions as well as limitations of data collection and representativeness. A commonly cited data limitation was the lack of representation of insecure clusters, as these often get dropped and/or more secure clusters are over sampled.

### Nutrition programme responses and multisectoral approaches

We found 20 eligible intervention studies matching inclusion criteria (Table 4). The studies ranged in publication dates from 1986 to 2017, 14 of which were published in or after 2013. However, all but two interventions were implemented prior to 2013. Most studies explicitly targeted children under 5 or 2 years of age and studies of household interventions specifically targeted women and young children. Interventions ranged from nutrition awareness and health promotion activities [62, 63, 75, 79], micronutrient deficiency treatment or prevention [50, 65–67, 80], malnutrition treatment or prevention [68, 70–72, 77, 78], and delivery or modelling of a package of community and facility-based interventions, including multisectoral approaches [64, 69, 74, 76, 81].

**Table 4:** Description of intervention studies

Source, type, geographic coverage, study type	Target group(s)	Intervention description	Intervention category	Multisectoral approach	Nutrition-related outcome categories and results	Lessons learned
Grant et al. (1986) [62] Published, database search Sub-national Programme evaluation	Children < 5 years	10–15 minutes of group education for waiting mothers by trained nurses to explain growth chart	Awareness	None	Literate and numerate mothers had significantly higher mean comprehension score than those who were not but no differential effect on nutrition status of their child; 62% of mothers understood the purpose and could distinguish good vs. bad weight, 49% understood upper line, 47% understood lower line, 45% understood space in between	Clinic staff time was consumed by explaining the chart, staff availability was the largest constraint on expanding services; authors cautioned activity expansion until randomised trial is conducted
Cheung, et al. (2003) [50] Published, database search Sub-national Case study	Households	Treatment for scurvy: 200 mg/day of vitamin C for 2 weeks for children and 1 g/day for 2 weeks for adults, including health education	Micronutrients	None	12 of 18 suspected scurvy cases were clinically confirmed (4 in children 3–5 years); over 3 months, the scurvy rate was 6.3% (4588 cases in population of 72,835) [severe level by WHO standards]; curative treatment of vitamin C tablets showed symptom reduction	Need field-friendly methods of confirming micronutrient-deficiency diseases; need to validate standard clinical case definition and include in endemic/outbreak-prone area surveillance; need to introduce and mainstream clinical micronutrient deficiency assessment and diagnosis into common assessment tools (i.e. nutrition surveys), train survey staff to identify micronutrient-deficiency diseases
Kim et al. (2008) [63] Published, database search Sub-national Programme evaluation	Households	Interactive electronic picture book, Afghan Family Health Book, to communicate public health messages on 17 topics: immunisation, micronutrients, WASH, diet, malaria, tuberculosis, acute respiratory infections, sexually transmitted disease, safety, first aid, mental health, female anatomy, birth spacing, breastfeeding and peripartum care	Awareness	None	Statistically significant improvements in knowledge on all health topics except female anatomy and sexually transmitted diseases; all users reported the Afghan Family Health Book to be too complicated and difficult to understand and CHW teachings were preferred	Scepticism of health education via electronic modes in areas where books are scarce and electronic devices are rare; interactive technology has potential to convey public health messages in such settings, although CHWs preferred
Grunewald et al. (2008) [64] Gray, hand search Sub-national Programme evaluation	Household, with a focus on mothers and children	20 FAO-executed projects; support to household food security, nutrition and livelihoods, with focus on piloting interventions and building MAIL capacity	Package of interventions	Agriculture and livelihood interventions with nutrition lens	Descriptive: lack of consistent monitoring and evaluation resulted in unmeasured indicators; those measured were mostly output indicators (e.g. number of trainings); used nutrition education booklets and posters with 9 key messages, trials of improved practices to improve household feeding practices and to test improved local IYCF and family recipes resulted in guide on improved feeding practices and recipes for Afghan children and mothers (3rd joint MAIL/MoPH publication)	Range of interventions left little time to test and accumulate sufficient data; questions remain for best target group; no thorough analysis of different models used, suitability of messages, no costs/benefits analysis; low land ownership/access to resources by women impeded project component implementation; practical livelihood support to increase production, diversify food production, food processing and conservation, and marketing should be provided with nutrition education
Manaseki-Holland et al. (2010) [65] Published, database search Sub-national RCT	Children 0–24 months	Supplementation of 100,000 IU of vitamin D3 (cholecalciferol), along with antibiotic treatment	Micronutrients	None	No significant difference in the mean number of days to recovery between two study arms; risk of a repeat episode within 90 days of supplementation was lower in intervention vs. placebo group (58%; relative risk 0.78; 95% CI 0.64–0.94); intervention group had longer duration to a repeat episode (72 days vs. 59 days; HR 0.71; 95% CI 0.53–0.95)	Repeat episodes of pneumonia could be reduced with a single high-dose oral vitamin D3 supplementation along with antibiotic treatment
Manaseki-Holland et al. (2012) [66] Published, database search Sub-national RCT	Children 1–11 months	Oral 100,000 IU (2.5 mg) vitamin D3 with placebo	Micronutrients	None	No significant difference between incidence of first or only pneumonia between vitamin D3 (0.145 per child-year, 95% CI 0.129–0.164) and placebo groups (0.137, 0.121–0.155)	Pneumonia incidence in infants was not reduced with quarterly doses of oral vitamin D3 supplementation
Aluisio et al. (2013) [67] Published, database search Sub-national RCT	Children 0–24 months	6 quarterly doses of oral vitamin D3 (cholecalciferol 100,000 IU or placebo)	Micronutrients	None	No significant difference in survival time to first diarrheal illness; incidence of diarrheal episodes were 3.43 (95% CI, 3.28–3.59) and 3.59 per child-year (95% CI, 3.44–3.76) in placebo and intervention arms, respectively	Authors do not recommend vitamin D3 supplementation to populations comparable to the one studied here given this study found no benefit for diarrheal illness prevention
Morikawa et al. (2013) [68] Published, database search Sub-national Programme evaluation	Children <5 years	Inpatient mother–child feeding centre that accommodates children <5 years with moderate malnutrition with psychosocial bonding support, SFP with monthly growth monitoring for 6 months after discharge until child reaches 85th percentile of their weight-for-height measure, lactating mothers also given food supplements at feeding centre with their child	Prevention and treatment of malnutrition	None	Observed significant and continuous gain in both weight and height of child during 6-month follow-up after discharge from feeding centre	Inpatient feeding focusing on building strong bonds between mothers and children should be evaluated for its impact on child development and nutrition
Munroe et al. [69] Gray, hand search National and sub-national Programme assessment (qualitative)	Households, health facility staff, policy-makers	Training on IYCF, micronutrients, health and hygiene for medical staff, CHS, CHW, health *shuras*, teachers, CDCs; training on family nutrition, including age-based diet requirements, food hygiene and healthy recipes, health staff trained on BF counselling and practices; nutrition education materials developed (BF, WASH, micronutrient guidelines), growth charts printed and training in growth monitoring; training on cooking sessions for complimentary feeding, growth monitoring, CMAM, community gardens, MUAC screenings; solar dehydration for food products, milk and potato processing, food processing and packaging training; nutrition material in national primary education curriculum; food-based dietary guidelines developed	Package of interventions	Agriculture interventions, non-health sector staff trained in growth monitoring, nutrition in primary school curriculum	Many programme output indicators, main nutrition indicators were MUAC-related, nutrition outcomes: GAM (MUAC <12.5 cm and/or bilateral oedema): 17.1%; MAM (MUAC 11.5–12.4 cm and no oedema): 10.3%, SAM (MUAC <11.5 cm and/or bilateral oedema): 6.8%	Interaction between agencies included an inception workshop and regular meetings but limited substantive collaboration in actual joint programming; many activities implemented in isolation by different IPs, making sustainability unlikely (i.e. study done by one IP on micronutrient deficiency not used by another IP with resultant mixed messaging about micronutrients, IPs implementing food security programmes using different models for the similar activities, etc.); where integration of nutrition and food processing did occur (guidelines developed and used by other agencies, food processing activities), activities were implemented directly by single team working together, with both nutrition and food security specialists; programme was geographically broad making it difficult to test an integrated model and measure attributable change
Ahmed et al. (2014) [70] Published, database search Sub-national Review of CMAM programmes	Children <5 years	CMAM, regular field monitoring and supportive supervision of nutrition activities	Malnutrition prevention and treatment	None	Case fatality rate in children with SAM admitted to hospitals is around 6%; SAM: 7.2%; MAM: 139%; SAM cases: 399,312	Constraints to implementing facility-based treatment of SAM: low coverage, lack of monitoring system, staff and space shortage, turnover of trained staff, lack of motivated staff; constraints to implementing CMAM treatment in communities: low coverage, monitoring, access to facility, lack of partnership; area for improvement: community mobilisation
Mayhew et al. (2014) [71] Published, database search Sub-national Programme evaluation comparing participants with non-participants	Children 0–24 months	cGMP: monitor weight of children 0–24 months to identify those not gaining adequate weight, give caretakers tools to aid children in ‘catch-up’ growth and promote optimal feeding practices, and create social change by mobilising caretakers to regularly weigh children <24 months and discuss appropriate food and feeding techniques	Malnutrition prevention and treatment	None	Where cGMP was implemented, a mean WFA Z-score was 0.3 Z-scores higher than among matched non-participants living outside cGMP programme catchment areas; those with initial WFA Z-score of <−2 experienced mean increase of 0.33 (95% CI 0.29–0.38)/session attended; those with baseline WFA Z-score >0 showed decrease of 0.19 (95% CI 0.22–0.15)/session attended	Potential to contribute to improving nutrition in underweight children who enter programme at less than 9 months of age and attend 50% or more sessions; authors suggest long-term evaluation to assess sustained growth in matched pairs of children up to 5 years of age and include more extensive inquiry into food security, wealth and other potentially confounding factors
Nasrat et al. (2014) [72] Gray, hand search Sub-national Programme assessment (qualitative)	EPHS/BPHS health facilities' clients	Nutrition component under BPHS and EPHS	Malnutrition prevention and treatment	None	Qualitative assessment of service delivery by key themes: staffing, training and capacity development (PND/PPHO understaffed, health staff not trained in nutrition services); management and support services (poor supervision and monitoring); service delivery (most facilities not offering complete nutrition service package, especially CHCs and BHCs)	Nutrition component is under-staffed and under resourced, optimal nutrition services not delivered through BPHS and EPHS
World Bank Group (2014) [73] Gray, hand search Sub-national Programme evaluation	Households, with focus on women and children 0-23 months	Nutrition and hygiene awareness pilot, part of Afghanistan Safety Nets Project (unconditional cash transfer), added as a soft conditionality to raise awareness: 2 educational sessions (beginning and end) with a small evaluation at midline to improve delivery between the two sessions; households received food packets and soap cakes; messages on handwashing at key points, IYCF (EBF/CF).	Awareness	Nutrition education with safety net programme	Participants appeared to understand importance of breastfeeding, but not when and how complementary food other than breastmilk should be introduced, appropriate complementary feeding was the most frequently misunderstood survey question; participants misunderstood messages on handwashing before key actions compared to after, especially importance of handwashing before feeding infants and children	No behaviour change data presented; more than two points of contact needed to ensure message retention; more focus on tools for targeting husbands and mothers-in-law due to their role as key influencers
Akseer et al. (2016) [74] Published, database search National and regional Observational study	Children < 5 years	Modelling of interventions with the Lives Saved Tool (LiST): EIBF (within 1 hour), 3 doses of DPT vaccine, measles vaccination, full immunisation of children, vitamin A supplementation, ORT and continued feeding for children with diarrhoea	Modelling package of interventions	None	EIBF: 53.6%, Q1: 52.1%; Q5: 54.3%; Vitamin A in past 6 months: 50.5%, Q1: 43.7%, Q5: 49.1%; Full immunisation: 17.6%, Q1: 13.2%, Q5: 19.4%; ORT: 45.8%, Q1: 47%, Q5: 54.3%	Significant variation in coverage and inequalities across various regions; composite intervention coverage lowest in most remote and isolated regions (Northern and Central Highlands) and highest in regions and provinces with major urban hubs (Nangarhar, Herat and Kabul)
JS Consultancy (2016) [75] Gray, hand search Sub-national Programme evaluation (qualitative)	Women who had a child ≤6 months	Brochures on best practices for newborn were provided to new parents with messages on EIBF, EBF for first 6 months, delayed bathing and recognising newborn complications for early care seeking	Awareness	None	No significant differences between baseline and endline in the proportion of women reporting any breastfeeding (96% vs. 94%) and EIBF; most women reported doing both	Focus on food consumption increased during pregnancy but not micronutrients, no details on ANC counselling on nutrition
Higgins-Steele et al. (2017) [76] Published, database search National Observational study	Children < 5 years	Using LiST tool: EBF <1 month, EBF 1–5 months, any BF 6–11 months, any BF 12–23 months, CF education only, CF supplementation and education, vitamin A supplementation, WASH, handwashing with soap, hygienic disposal of children's stools, pentavalent vaccine, pneumococcal vaccine, rotavirus vaccine, measles vaccine, injectable antibiotics, ORS, antibiotics for dysentery, zinc for diarrhoea, oral antibiotics for case management of pneumonia, therapeutic feeding for severe wasting, MAM treatment	Modelling package of interventions	WASH interventions included in the modelling	71% reduction in child deaths due to diarrhoea and pneumonia between 2016 and 2020 (for diarrhoea, a 85% reduction; for pneumonia, a 63% reduction) compared to 47% reduction in the moderate scenario (for diarrhoea, a 35% reduction; for pneumonia, a 63% reduction)	Better modelling tools are needed to adequately capture impact of nutrition on childhood mortality, investment is needed to strengthen CHW cadre, expand coverage of immunisations
Venkataramani et al. (2017) [77] Published, database search National Observational study	Children < 5 years	IMCI screening	Malnutrition prevention and treatment	None	Primary outcome was an assessment index measuring the healthcare provider’s adherence to selected IMCI screening tasks; visits with any IMCI-related complaint were associated with higher assessment indices than visits with no IMCI-related complaints	Presenting complaints are an important factor in providers adhering to the IMCI assessment algorithm; children who present with only non-IMCI complaints may be at risk for not being screened for critical IMCI conditions
Mansoor et al. (2017) [78] Published, hand search National Programme evaluation	Children < 5 years	Introduction and scale-up of IMCI at primary healthcare facilities	Malnutrition prevention and treatment	None	On average, 5.4 of 10 main assessment tasks were performed during paediatric examination; more than half were assessed for three main symptoms of cough, diarrhoea and fever; 28% (*n* = 30) of children <2 years of age were assessed for feeding practices; 34% (*n* = 60) were weighed and checked against growth chart	IMCI training not fully scaled to cover all health workers, healthcare providers trained on IMCI more likely than untrained providers to conduct a systematic assessment of a child’s condition
Pedersen S, et al. (2016) Gray, hand search Sub-national Programme evaluation (qualitative)	Children <5 years, mothers	Package of community and facility-based interventions that provide preventive and curative health and nutrition services; established and trained FHAGs, CHWs and HF staff on IYCF messages; IYCF counselling; Positive Deviance Hearth; cGMP; community WASH groups; VIP latrines; dietary diversity awareness; home garden support and training for women, poultry livelihood training and distribution; MNP distribution; strengthen IPD/OPD SAM; CMAM/IYCF training for nutrition nurses; CME/CNE nutrition SOP training	Package of interventions	Agriculture interventions (home gardens and chickens for women)	Of the 18 MUNCH interventions, 10 had targets listed in the annual work plans; targets were met for Timed and Targeted Counselling training and exceeded for latrine building, establishing IPD/OPD SAM Centres, distribution of MNP, distribution of chickens, and establishing home gardens; three interventions, gender equality training, mHealth (mobile health) training, and IYCF message training, missed their targets. Positive Deviance Hearth achieved 50% of its target number of children achieving minimum weight	FHAGs, Positive Deviance Hearth and WASH groups were particularly important to establishing good practices in the community; these interventions demonstrate value of community driven action
Global Alliance for Improved Nutrition (2017) Gray, hand search National Evaluation	Households	National salt iodisation and fortification of wheat flour and oil	Micronutrients	Micronutrient fortification in foods	Awareness of fortification was low: 22% of households reported hearing about fortified foods, 35% in Kabul compared to 33% in other urban areas and 20% in rural areas; level of fortification was inconsistent with national standards: 2% of salt brands, 4% of oil brands and 10% of wheat flour brands were fortified within the standard range; 71% of salt brands and 51% of wheat flour brands were partially fortified but only 35% of oil brands were fortified at all	High potential for impact from large-scale fortification of salt and oil; potential for wheat flour is lower; further exploration needed to assess feasibility of targeting small-scale producers; for all food vehicles, monitoring, regulation and enforcement are critical to improve the level of fortification, for both domestic and imported products; future research should assess nutrient contribution from fortified foods and total intake of nutrients from all dietary sources to see if dietary nutrient gap is filled through fortification efforts

*ANC* antenatal care, *BF* breastfeeding, *BHC* basic health center, *BPHS* basic package of health services, *CDC* community development council, *CF* complementary feeding, *CHC* comprehensive health center, *CHS* community health supervisor, *CHW* community health worker, *cGMP* Community growth monitoring and promotion, *CMAM* community-based management of acute malnutrition, *DPT* diphtheria, *EBF* exclusive breastfeeding, *EIBF* early initiation of breasfeeding, *EPHS* essential package of hospital services, *FHAG* family health action group, *GAM* global acute malnutrition, *HF* health facility, *IMCI* integrated management of childhood illness, *IPD* inpatient department, *IYCF* infant and young child feeding, *MAIL* Ministry of Agriculture, Irrigation, and Livestock, *MAM* moderate acute malnutrition, *MoPH* Ministry of Public Health, *MNP* micronutrient powder, *MUAC* Mid-Upper Arm Circumference, *MUNCH* Maternal and Under-Five Nutrition and Child Health, *ORS* Oral rehydration solution, *ORT* oral rehydration therapy, *OPD* outpatient department, *PND *Public Nutrition Department, *PPHD* Provincial Public Health Department, *SAM* severe acute malnutrition, *SFP* supplementary feeding programme, *SOP* standard operating procedure, *WASH* water, sanitation, and hygiene, *WFA* weight-for-age

#### Nutrition awareness and health promotion

Nutrition awareness is often promoted through campaigns on different topics such as breastfeeding, hygiene, growth monitoring and complementary feeding at group education sessions [63, 71, 81], training for women and households participating in kitchen gardens and select agriculture activities [81], and other targeted projects. These tend to be topic and agenda specific by project. Almost all interventions included an education component, with four studies also focused on raising awareness and promoting health and nutrition messages. These were done through group education for mothers on child growth charts [62], interactive electronic books with public health messages [63], a nutrition and hygiene awareness pilot as part of an unconditional cash transfer project [79], and brochures on IYCF best practices for newborns [75]. These nutrition awareness programmes all included mothers of young children as the target audience, despite pilot study findings of additional benefit in targeting men or mothers-in-law (or other influential family members) with tailored messages [79]. All studies reported some improvement in knowledge levels of participants, but these were either qualitative feedback from participants or sample sizes were generally small and statistical tests were not often used to compare pre- and post-intervention changes. One study noted that understanding the importance of breastfeeding was high but the timing for complementary food introduction and handwashing before feeding children were frequently misunderstood [79]. Only one study assessed self-reported IYCF practices and found no differences between baseline and endline in early initiation of breastfeeding [75].

#### Prevention and treatment of micronutrient deficiencies

There were four types of programmes designed to reduce micronutrient deficiencies in Afghanistan – food diversification, micronutrient supplementation, food fortification and micronutrient deficiency disorder treatment [2]. Four studies evaluated micronutrient deficiency disorder treatment, specifically vitamin C to treat scurvy [50], and micronutrient supplementation, specifically vitamin D3 to reduce the duration and occurrence of pneumonia [65, 66], and to decrease the incidence of diarrhoea in children under 5 years [67]. Vitamin D3 was found to be ineffective for reducing the incidence of either diarrhoea or pneumonia in the studied population but was effective for reducing recurrent episodes of pneumonia when taken with antibiotics.

One study assessed national food fortification coverage and found that the level of fortification was below national standards: only 2% of salt brands, 4% of oil brands and 10% of wheat flour brands were fortified within the standard range [80]. Household consumption of fortified food was also low; only 22.1% of households consumed fortified salt, 30.1% consumed fortified oil and 18.6% consumed fortified wheat flour [80]. The low availability and consumption of fortified wheat flour is likely due to production and milling through small-scale systems that do not provide fortification, used by more than 80% of rural households [82].

At the facility level, micronutrient supplementation is provided by midwives, vaccinators and doctors. Midwives provide iron and folic acid supplements for pregnant and lactating women, vaccinators provide vitamin A supplements for children 6–59 months of age, and doctors provide iron for low birth-weight babies and vitamin C for populations at high risk. An assessment of the Basic Package of Health services (BPHS) and the Essential Package of Hospital Services (EPHS) nutrition components found that, while health workers are tasked with promoting fortified food consumption, such as iodised salt, and community health workers (CHWs) are tasked with including discussions on fortified foods at community health *shura* (community leadership committee) meetings, many households likely do not have access to fortified flour and oil [72]. Additionally, CHWs are supposed to provide micronutrient powders for home-based fortification for all children under 2 years of age; however, micronutrient powders are not on the essential drug list for health posts in the BPHS. Additionally, the BPHS essential drug list does not include multi-micronutrient vitamins at the health post (community) level. Thus, these supplies are often not available at community level, unless supplied via an external project.

#### Prevention and treatment of malnutrition and childhood illnesses

The Integrated Management of Childhood Illness (IMCI) programme was adopted globally as a strategy to reduce morbidity and mortality in children under 5 years of age [83]. IMCI was introduced to Afghanistan in 2003, with implementation across the country as part of the BPHS. IMCI includes case management of acute respiratory infection, diarrhoea, ear problems, malaria and other febrile illnesses, measles, malnutrition, and anaemia and provides oral drugs and immunisations [84]. Challenges to ensuring the high quality of IMCI services have been documented and primarily relate to the availability of doctors, the receipt of standard IMCI training for health workers, large patient volumes, poor supervision and limited availability of clinical guidelines [85, 86]. The IMAM is the main malnutrition prevention and treatment strategy. Initially the management of acute malnutrition started as an emergency programme at the community level, introduced as CMAM in 2010. However, to address nutrition prevention and treatment needs comprehensively, the MoPH decided to scale up the management of acute malnutrition through the BPHS and EPHS and shift the focus from emergency services to development and sustainable programming, predominantly at the facility level [33]. Four studies assessed CMAM [70], IMAM [77, 78], and the delivery of nutrition services through the BPHS and EPHS [72]. Across these studies, major constraints to preventing and treating malnutrition at facility and community levels included low nutrition service coverage, shortage of staff, poorly trained staff, and poor supervision and monitoring. Venkataramani et al. [77] found that children who present with IMCI-related complaints (fever, diarrhoea or cough) influence a provider’s adherence to the IMCI screening protocol, meaning that children with non-IMCI complaints may be at risk of not being screened for critical but less acutely symptomatic IMCI conditions such as anaemia and malnutrition. Mansoor et al. [78] also found that provider adherence to IMCI algorithms varied and, on average, 5.4 of 10 main assessment tasks were performed during examination of a child. Overall, the nutrition component of the BPHS and EPHS was found to be under-staffed and under-resourced, with optimal nutrition services not delivered [72]. Morikawa et al. [68] evaluated a programme of an inpatient mother–child feeding centre with psychosocial bonding support and supplementary feeding for both mother and child, and growth monitoring for up to 6 months after discharge. They observed significant and continuous improvements in both weight and height during the 6-month follow-up but did not evaluate the impact of an integrated inpatient feeding programme focused on psychosocial bonding on child development and nutrition over the longer term. Mayhew et al. [71] observed the potential impacts of a community growth monitoring programme to aid caretakers in identifying children who had not gained adequate weight and to promote optimal feeding practices for ‘catch-up’ growth. They recommended a long-term evaluation of the programme using matched pairs of children; however, to date, such a study has not been conducted though similar programmes continue to be implemented.

#### Delivery or modelling of a package of community and facility-based interventions, including multisectoral approaches

Packages of community and facility-based nutrition-related interventions implemented in Afghanistan often included agriculture and livelihoods programmes designed with a nutrition lens, with three large-scale multisector projects to date. In addition to nutrition-specific interventions (e.g. IYCF, CMAM, hygiene awareness), nutrition-sensitive interventions in these packages included the provision of seeds, fertiliser and tools for farming [69], kitchen gardens [69, 81], improved latrines [81], dietary diversity awareness [69, 81], and small-scale livestock for poultry and dairy production [64, 69, 81]. An assessment of 20 FAO-executed projects [64] found a lack of consistent monitoring and evaluation measures, which impeded accumulation of sufficient data to show meaningful programmatic changes. While other large-scale intervention packages were found to meet their implementation targets [81], these packages of multisectoral interventions were limited in testing combinations of interventions, their delivery approaches and understanding their effectiveness on improving nutrition outcomes. A common thread among multisector programmes was the dependence upon and value of community actors such as volunteer CHWs, Family Health Action groups, community development councils/*shuras* and WASH groups. These community level actors are crucial for mobilisation efforts and for creating demand for services. Integration of nutrition activities occurred through guidelines developed and used by other agencies, which incorporated nutrition topics into primary education curricula or food processing activities. However, these were implemented directly by a single team comprised of both nutrition and food security specialists working together.

Two studies used the Lives Saved Tool to model the impact of a combination of IYCF interventions, community-based health and nutrition interventions, and WASH on improving child health and nutrition outcomes. Akseer et al. (78) and Higgins-Steele et al. ( [76]) found significant regional variations in coverage and inequalities and that better modelling tools are needed to adequately capture the spectrum of nutrition-sensitive and nutrition-specific interventions and their impacts on child mortality.

## Discussion

This scoping review synthesised information from 18 policies and strategies, 45 data sources and reports, and 20 intervention evaluations relevant to improving nutrition among children in Afghanistan. Key findings from this scoping review include the following: (1) a shift toward multisectoral efforts to address malnutrition at the policy level has started but nutrition-specific and nutrition-sensitive interventions are not yet uniformly delivered or integrated at the community level; (2) political will to improve household nutrition status is reflected in increased government and donor investments in nutrition-sensitive and nutrition-specific programmes through a combination of small- and large-scale interventions between 2004 and 2013, but there is limited evidence for interventions that effectively contributed to decreased stunting prevalence; and (3) many data sources capturing nutrition, food security and WASH indicators are available, but efforts are needed to standardise indicator definitions and longitudinal nutrition surveys should be considered to assess change over time and ensure relevance to policy needs.

Aligning with the global momentum for improving nutrition outcomes through a combination of evidence-based nutrition-specific and nutrition-sensitive approaches, Afghanistan has made efforts to prioritise nutrition and food security and, specifically, to address poor child nutrition indicators. However, Afghanistan’s progress is undermined by the lack of or weak programmatic efforts to generate demand for nutrition and food interventions, often due to the reliance on volunteers that may not be incentivised to create the necessary level of demand. For example, one nutrition and hygiene promotion programme was pilot-tested among a subset of communities that were a part of a larger unconditional cash transfer project. Evaluators mentioned that participation and interest in the awareness campaigns were high, partly because households had the impression that they might become eligible for the cash transfer programme [79]. Similarly, SMART and SQUEAC assessments found that one of the largest challenges was communities’ lack of recognition of malnutrition as a major health concern and their low awareness of malnutrition programmes offered by health facilities [87]. Recognising the need for more innovative forms of demand creation for nutrition information and services, programmes such as the Community-Based Nutrition Package (CBNP), Maternal and Child Health (MCH) handbook, and Community-Led Total Sanitation have recently been introduced and nutrition counsellors have been added as part of BPHS facility staff. The CBNP programme was developed as a comprehensive minimum service package targeting high-impact interventions in the areas of nutrition, WASH and food security for pregnant women and families with children under 24 months of age. It has been implemented in 15 provinces and a recent process evaluation revealed that the CBNP provided adequate quantity and quality of equipment and supplies and refresher training for CHWs [88]. The MCH handbook was piloted in one district each of Kabul and Nangarhar provinces and distributed to pregnant women or women with children under 24 months of age and promotes all stages of maternal, newborn and child health, including health promotion, hygiene and growth monitoring. Based on positive assessment results of the handbook’s reach and use, national scale-up over 3 years is planned [89]. Community-Led Total Sanitation has been adopted by the government to mobilise communities to be open defecation-free and to motivate households to improve their traditional latrines; over 1000 communities have remained open defecation-free since 2010 [90]. Progress has also been made towards staffing BPHS facilities with a nutrition counsellor; about 87% of the planned HFs have been staffed with a nutrition counsellor, of whom 75% have received the national training package [91].

Ten high impact nutrition-specific interventions across the lifecycle through the first 1000 days were modelled globally to 90% coverage, which resulted in reducing stunting prevalence by 20% and wasting prevalence by 60% [3]. These ten interventions have been implemented in Afghanistan to some degree and include periconceptual folic acid supplementation, maternal balanced energy protein supplementation, maternal calcium supplementation, multiple micronutrient supplementation in pregnancy, promotion of breastfeeding, appropriate complementary feeding, vitamin A administration and preventive zinc supplementation in children 6–59 aged months, and screening for and management of severe acute malnutrition and MAM [3]. Targeting is essential for implementation of high-impact nutrition interventions at scale. Because most interventions in Afghanistan containing knowledge or behaviour change components target mothers and young children (either under 5 years of age or 24 months of age, shown in Table 4), future programmes should consider tailoring messages to men and ensuring greater male engagement as they are the normative decision-makers for healthcare access, household expenditures and foods cultivated within the household. Further, not all pregnant women, mothers and young children require all of the above-mentioned interventions – communities, women and children should be more selectively targeted based on vulnerability and need, allowing for greater cost effectiveness and efficiencies when programming at scale [92]. Multisectoral adaptive management capacity should be strengthened for effective targeting to occur.

Several high level multisectoral policy and strategy documents have been commissioned by the government; government and international partners have supported large-scale nutrition, food security and WASH programmes, though most of these have focused on the health systems supply side and the health sector has worked to strengthen and integrate the nutrition package delivered through its key service delivery platform, the BPHS/EPHS. Given the paucity of evidence in the peer-reviewed literature regarding nutrition interventions in Afghanistan and similar fragile settings, we find current value in grey literature and potentially disagree with excluding this source through systematic reviews or more limited scoping reviews. In countries like Afghanistan, grey literature reports may constitute a substantial amount of contextually relevant data available and accessible to guide policy and programme design. This is likely true for integrated, multisector approaches for improving nutrition, given that evaluation of integrated programmes is complex and methods used are often not appropriately designed [93]. While greater efforts are needed to improve the quality of research in this setting, rapid and well-defined approaches for assessing the whole body of literature – peer-reviewed publications and grey literature documents – and synthesising evidence for decision-making is needed.

Impacts on nutrition outcomes were not evaluated in any of the included studies due to relatively brief implementation timelines, coverage areas and lack of appropriate study designs. Most evaluations were largely descriptive and focused on programme outputs and targets achieved. Only BPHS and EPHS nutrition services and fortification programmes were designed to reach a national scale but quality, coverage, scope and compliance issues have been reported [72]. All other interventions were implemented in small programme areas, lacking sufficient coverage and scale. Multisector approaches particularly faced challenges of reaching sufficient coverage as they often included a package of interventions across food security, livelihoods and health but were implemented independently of one another. Parallel implementation or improved coordination of multisectoral efforts would be more effective than independent programmes in regions with high malnutrition rates to obtain full-service coverage. Questions remain regarding the most appropriate target group(s), analytic approaches for data from the different delivery models used, suitability of messages, and calculations of costs and benefits. At the policy level, while adequate multisector plans and strategies have been developed, implementation and appropriate coordination mechanisms have delayed their full functionality. Further, at the provincial and field implementation level, coordination structures across ministry sectors are non-existent or remain nascent. The establishment of the AFSeN Secretariat is one high level coordination structure that has been expanded to 12 provincial AFSeN committees. There is currently some uncertainty surrounding continuity and the executive office sponsor, but the provincial AFSeN committees continue policy and programming oversight.

This scoping review shows a breadth of nutrition programme and policy data in Afghanistan. However, measures of nutrition-sensitive and nutrition-specific outcomes, practices and knowledge areas are inconsistent over time and studies are mainly cross-sectional in design. Repeated national cross-sectional surveys remain the single source of national nutrition status indicators, such as the most recent AHS 2018, which provides updated information on child nutrition status indicators. The AHS 2018 as compared to the NNS 2013 shows a slight improvement in moderate stunting (36.6% vs. 40.9%), severe stunting (17.3% vs. 20.9%), and no improvement in reported exclusive breastfeeding of children under 6 months (57.5% vs. 58.4%). However, these variations in study design and key indicators and their definitions preclude meta-analyses. For example, exclusive breastfeeding is a standard IYCF indicator, but there was substantial variation in duration (Table 3) that departs from defined international standards used by WHO and Demographic Health Surveys [34, 36, 39, 41]. Most studies are unable to measure stunting, a main impact indicator, across a shorter time period as it is not sensitive to small changes over time [94] and there is a lack of objective interim measures to assess nutritional status. High levels of stunting are associated with poor socioeconomic conditions; however, improvements in nutrition outcomes over time may not necessarily indicate improved socioeconomic conditions for that specific cohort of children under 5 years of age as they are now school aged [95]. For this reason, longitudinal nutrition studies that monitor linear growth trajectories of children into their early school years would provide a better understanding of intergenerational associations, social differentials and environmental influences [96].

More recent secondary analyses of available data have shown decreasing trends in optimal breastfeeding practices [23] and that socioeconomic inequities are associated with impaired complementary feeding practices [60]. Analysis has also shown positive associations between irrigation, garden plots and dietary diversity, suggesting the need for integration of market strategies, household food production and nutrition education [61]. Current evidence also shows that there are significant district-level geographic disparities in nutrition indicators among children [59]. Improved access to water and sanitation was also a strong predictor of child and maternal nutritional status [59]. Integrated programmes in Afghanistan have not been rigorously evaluated, analysed for combined effects on nutritional status or assessed for optimal implementation strategies. Further research is needed to understand reasons for these declining trends. Implementation evidence is also needed to aid policy and programmes on effective integration of nutrition, food security and WASH. This information should be used for programme planning and targeting beneficiaries.

The all-inclusive approach to large scale programmes allows for piloting and introducing a number of new interventions at once, but testing, refining and identifying what works as a single intervention or as an integrated approach is not often assessed. Small-scale surveys, such as the SMART and SQUEAC surveys, are conducted by implementing partners, but the extent to which data from these surveys have been used to continuously inform and improve their programmes is unknown. Follow-up surveys were not identified nor included in this study.

This scoping review has some limitations. While we did not restrict by time, the exclusion of non-English language documents may have excluded relevant documents. The scoping review methodology allowed for a broad search strategy, which we refined iteratively to maximise eligible source identification. However, some relevant grey literature documents may not have been identified for this review. We were surprised at the minimal inclusion of WASH interventions in eligible reports, considering the long-standing and scaled WASH efforts in Afghanistan. While we aimed to keep our search and inclusion strategy broad, studies were only eligible if relevant nutrition-related indicators, including hygiene promotion, were measured. However, water and sanitation activities may measure outputs of facilities only, which were beyond the scope of our review. We did not assess the quality of the studies included in this review. Evidence for the effectiveness of the evaluated interventions is limited, resulting in studies from Afghanistan being excluded in systematic literature reviews of nutrition intervention efficacy. We attempted to ensure that our inclusion criteria were broad for this scoping review, but only 12 peer-reviewed studies and 8 evaluations from the grey literature were eligible for inclusion. It is notable that 4 grey literature reports assessing nutrition-related programmes were not included because they were not fully conducted by a third party [97–100]. Donors and implementing partners should ensure that programme evaluations are planned from the beginning with adequate funds allocated.

## Conclusion

The government of Afghanistan, international donors and implementing partners have made important investments in introducing nutrition-specific and nutrition-sensitive services across the country. Progress in addressing malnutrition should be accelerated with a focus on integrated multisectoral action.

## Supplementary information

**Additional file 1.**

## Data Availability

Not applicable. All data reported are publicly available in studies included in this scoping review.

## Abbreviations

ADHS: Afghanistan Demographic and Health Survey
AFSeN: Afghanistan Food Security and Nutrition Agenda
AHS: Afghanistan Health Survey
ALCS: Afghanistan Living Conditions Survey
ANDS: Afghanistan National Development Strategy
BPHS: basic package of health services
CBNP: community-based nutrition package
CHWs: community health workers
CMAM: community-based management of acute malnutrition
EBF: exclusive breastfeeding
EPHS: essential package of hospital services
IMAM: integrated management of acute malnutrition
IMCI: integrated management of childhood illness
IYCF: infant and young child feeding
MAIL: Ministry of Agriculture, Irrigation, and Livestock
MAM: moderate acute malnutrition
MICS: Multiple Indicator Cluster Survey
MoPH: Ministry of Public Health
MUAC: Mid-Upper Arm Circumference
NGO: non-governmental organisation
NNS: National Nutrition Survey
NRVA: National Risk and Vulnerability Assessment
SDG: Sustainable Development Goal
SMART: Standardized Monitoring and Assessment of Relief and Transitions
SQUEAC: Semi-Quantitative Evaluation of Access and Coverage
WASH: Water Sanitation and Hygiene
