# Supplementary material for: Multisector nutrition gains amidst evidence scarcity: scoping review of policies, data and interventions to reduce child stunting in Afghanistan
Source: Health Res Policy Syst. 2020 Jun 11;18:65. doi: 10.1186/s12961-020-00569-x (PMC7291673; doi:10.1186/s12961-020-00569-x)
Supplement: Supplementary file 1 — Additional file 1. [file 12961_2020_569_MOESM1_ESM.docx]

**Supplement: Search Strategy**

**PubMed**

(“child nutrition” OR “infant and young child feeding” OR  child nutrition sciences[Mesh] OR  “infant and young child feeding”  OR Prenatal Nutritional Physiological Phenomena[Mesh] OR child nutritional physiological phenomena[Mesh] OR  ((nutritional status[Mesh] OR "nutrition sensitive" OR “nutrition” OR nutritional requirements[Mesh] OR "nutrition specific" OR “undernutrition” OR “malnutrition”  OR  hunger OR nutrition assessment[Mesh] OR nutrition surveys[Mesh] OR nutrition policy[Mesh] OR  “nutrition during pregnancy” OR “maternal nutrition” OR “maternal undernutrition” OR “maternal malnutrition” OR “Maternal Nutritional Physiological Phenomena”[Mesh] OR “maternal iron supplementation” OR “dietary supplements during pregnancy” OR Food Labeling[Mesh] OR “exclusive breastfeeding” OR “feeding practice” OR dietary diversity OR MUAC OR “middle upper arm circumference” OR stunting OR wasting) AND (child OR children OR infant)) AND Afghanistan[Mesh]

Limited to Human

Results:  58 refs

**Popline**

(child* OR infant OR infants) AND (nutrition OR malnutrition OR undernutrition OR hunger OR food OR "nutrition sensitive" OR "nutrition specific" OR "nutritional status")

OR

(stunting OR wasting OR "exclusive breastfeeding" OR "feeding practice" OR MUAC OR "middle upper arm circumference" OR "food security" OR "food insecurity" OR "nutrition security" OR "nutrition insecurity" OR "food and nutrition security" OR "food and nutrition insecurity" OR "food supply" OR "dietary diversity")

AND

Afghanistan

Results:  79 refs

**Embase**

(“child nutrition” OR “infant and young child feeding” OR  child nutrition sciences OR  “infant and young child feeding”  OR Prenatal Nutritional Physiological Phenomena OR child nutritional physiological phenomena OR  ((nutritional status OR "nutrition sensitive" OR “nutrition” OR nutritional requirements OR "nutrition specific" OR “undernutrition” OR “malnutrition”  OR  hunger OR nutrition assessment OR nutrition surveys OR nutrition policy OR  “nutrition during pregnancy” OR “maternal nutrition” OR “maternal undernutrition” OR “maternal malnutrition” OR Maternal Nutritional Physiological Phenomena OR “maternal iron supplementation” OR “dietary supplements during pregnancy” OR Food Labeling OR “exclusive breastfeeding” OR “feeding practice” OR dietary diversity OR MUAC OR “middle upper arm circumference” OR stunting OR wasting) AND (child OR infant)))AND Afghanistan

Limited to Embase

Results: 17 refs

**Global Health, Academic Search Premier, CINAHL Plus with Full Text, EconLit, Education Full Text (H.W. Wilson), Environment Complete, ERIC, GreenFILE, Middle Eastern & Central Asian Studies**

(“child nutrition” OR “infant and young child feeding” OR  child nutrition sciences OR  “infant and young child feeding”  OR Prenatal Nutritional Physiological Phenomena OR child nutritional physiological phenomena OR  ((nutritional status OR "nutrition sensitive" OR “nutrition” OR nutritional requirements OR "nutrition specific" OR “undernutrition” OR “malnutrition”  OR  hunger OR nutrition assessment OR nutrition surveys OR nutrition policy OR  “nutrition during pregnancy” OR “maternal nutrition” OR “maternal undernutrition” OR “maternal malnutrition” OR Maternal Nutritional Physiological Phenomena OR “maternal iron supplementation” OR “dietary supplements during pregnancy” OR Food Labeling OR “exclusive breastfeeding” OR “feeding practice” OR dietary diversity OR MUAC OR “middle upper arm circumference” OR stunting OR wasting) AND (child OR infant))) AND Afghanistan

Results:  163 refs, 135 when imported into Endnote - dups

**Web of Science**

26 Sept 2018

((child nutrition OR infant and young child feeding OR child nutrition sciences OR infant and young child feeding OR Prenatal Nutritional Physiological Phenomena OR child nutritional physiological phenomena OR ((nutritional status OR "nutrition sensitive" OR nutrition OR nutritional requirements OR "nutrition specific" OR undernutrition OR malnutrition OR hunger OR nutrition assessment OR nutrition surveys OR nutrition policy OR nutrition during pregnancy OR maternal nutrition OR maternal undernutrition OR maternal malnutrition OR Maternal Nutritional Physiological Phenomena OR maternal iron

supplementation OR dietary supplements during pregnancy OR Food Labeling OR exclusive breastfeeding OR feeding practice OR dietary diversity OR MUAC OR middle upper arm circumference OR stunting OR wasting) AND (child OR children OR infant))) AND Afghanistan)

Results: 46 refs
